# Neutrophil-Derived Microvesicle Induced Dysfunction of Brain Microvascular Endothelial Cells In Vitro

**DOI:** 10.3390/ijms20205227

**Published:** 2019-10-22

**Authors:** Anjana Ajikumar, Merete B. Long, Paul R. Heath, Stephen B. Wharton, Paul G. Ince, Victoria C. Ridger, Julie E. Simpson

**Affiliations:** 1 Sheffield Institute for Translational Neuroscience, University of Sheffield, Sheffield S10 2HQ, UK; AAjikumar1@sheffield.ac.uk (A.A.); p.heath@sheffield.ac.uk (P.R.H.); s.wharton@sheffield.ac.uk (S.B.W.); p.g.ince@sheffield.ac.uk (P.G.I.); 2 Department of Infection Immunity and Cardiovascular Diseases, University of Sheffield, Medical School, Sheffield S10 2RX, UK; mlong3@sheffield.ac.uk (M.B.L.); v.c.ridger@sheffield.ac.uk (V.C.R.)

**Keywords:** neutrophils, microvesicles, blood brain barrier, endothelial cells, TEER, gene expression, permeability

## Abstract

The blood-brain barrier (BBB), composed of brain microvascular endothelial cells (BMEC) that are tightly linked by tight junction (TJ) proteins, restricts the movement of molecules between the periphery and the central nervous system. Elevated systemic levels of neutrophils have been detected in patients with altered BBB function, but the role of neutrophils in BMEC dysfunction is unknown. Neutrophils are key players of the immune response and, when activated, produce neutrophil-derived microvesicles (NMV). NMV have been shown to impact the integrity of endothelial cells throughout the body and we hypothesize that NMV released from circulating neutrophils interact with BMEC and induce endothelial cell dysfunction. Therefore, the current study investigated the interaction of NMV with human BMEC and determined whether they altered gene expression and function in vitro. Using flow cytometry and confocal imaging, NMV were shown to be internalized by the human cerebral microvascular endothelial cell line hCMEC/D3 via a variety of energy-dependent mechanisms, including endocytosis and macropinocytosis. The internalization of NMV significantly altered the transcriptomic profile of hCMEC/D3, specifically inducing the dysregulation of genes associated with TJ, ubiquitin-mediated proteolysis and vesicular transport. Functional studies confirmed NMV significantly increased permeability and decreased the transendothelial electrical resistance (TEER) of a confluent monolayer of hCMEC/D3. These findings indicate that NMV interact with and affect gene expression of BMEC as well as impacting their integrity. We conclude that NMV may play an important role in modulating the permeability of BBB during an infection.

## 1. Introduction

The blood brain barrier (BBB) plays an integral role in maintaining central homeostasis by acting as a physical barrier between the central nervous system (CNS) and the periphery. It is formed by capillary endothelial cells, the basement membrane and surrounding pericytes and astrocyte endfeet, this highly specialised structure exhibits high transendothelial electrical resistance (TEER) and restricts the paracellular movement of molecules. Disruption, with increased permeability, of the BBB is a feature of many neurodegenerative diseases, including Alzheimer’s disease (AD), vascular dementia (VaD), and multiple sclerosis [1,2,3].

Continuous peripheral activation of the immune system during systemic infection can affect the CNS and activate microglia, the resident immune cells [4]. Clinical studies have reported that peripheral infection and systemic inflammation lead to the progression of cognitive decline in AD [5,6,7]. Chronic inflammatory conditions such as atherosclerosis [8,9], obesity [10], and periodontitis [11] are either risk factors for and/or associated with late-onset AD. Animal models have also demonstrated that peripheral activation of immune cells by inflammatory stimuli such as lipopolysaccharide (LPS) can impact BBB permeability, increasing microglial activation [12,13,14,15,16]. Together, these studies support a role for systemic inflammation in modulating BBB function and increasing neuroinflammation within the CNS.

Neutrophils are the most abundant white blood cells in the circulation and act as the first line of defense through various mechanisms, including the rapid production of reactive oxygen species, phagocytosis, degranulation and release of extracellular traps. Neutrophil depletion in transgenic murine models of AD reduces AD-like pathology by reducing amyloid-β deposits and improves cognition in mice [17]. Patients with dementia have significantly higher levels of circulating neutrophils than non-neurological controls [18], and their activation status correlates with AD progression [19]. While these studies indicate a role for neutrophils in AD, neutrophil extravasation is not routinely associated with AD pathology, and the effect of neutrophils on the BBB is currently unknown.

There is increasing evidence that cells use plasma membrane-derived microvesicles (MV) to communicate with distant target cells [20,21]. Neutrophils produce low levels of neutrophil-derived microvesicles (NMV) under normal physiological conditions [22]. However, elevated levels of NMV have been reported under inflammatory conditions [22,23,24]. NMV vary in size, ranging from 100 to 1000 nm in diameter, and have a double layered membrane [25,26]. They contain a range of inflammatory proteins, mRNA, microRNA and adhesion molecules such as CD11a and L-selectin [27,28]. NMV have been shown to impair the integrity of other endothelial cells such as human coronary arterial endothelial cells (HCAEC) [29], human umbilical vein endothelial cells (HUVEC) [30] and other myeloid cells [31], but their effects on BMEC are unknown.

The current study tested the hypothesis that in response to systemic infection NMV are internalized by BMEC, altering their integrity. Specifically, the study aimed to determine whether NMV interact with BMEC, their mechanism of internalization and NMV-induced transcriptomic changes in brain endothelial cells.

## 2. Results

### 2.1. Characterization of NMV

**Figure 1:**
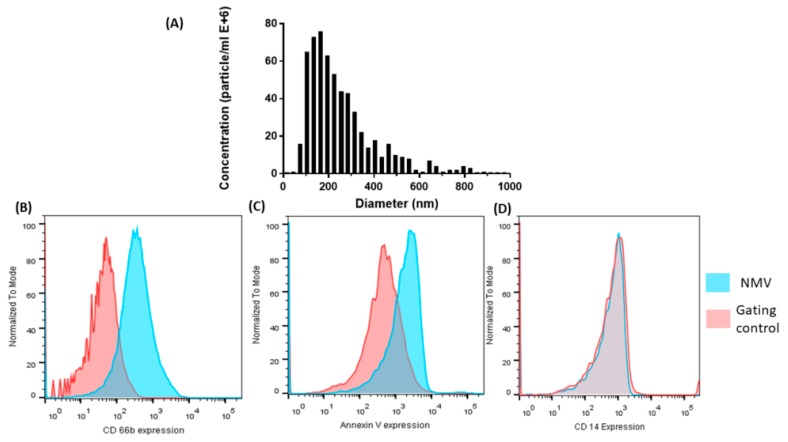
Characterization of neutrophil-derived microvesicles (NMV) internalization by hCMEC/D3. (**A**) NMV size distribution were measured using ZetaView nanoparticle tracking analysis. NMV express (**B**) the neutrophil surface antigen CD66b and (**C**) annexin V, but not (**D**) CD14 (monocytes), as detected by flow cytometry. Isotype control was used to gate for CD66b and CD14 positivity, unlabeled NMVs were used to set the gate for Annexin V positivity. Histograms are representative of three individual neutrophil isolations.

NMV isolated from the blood of healthy volunteers were analyzed using ZetaView nanoparticle tracking analysis (NTA) revealed they were heterogenous with a mean size of 226 nm (Figure 1A), confirming previous characterization studies carried out in our laboratory [29]. The majority of NMV were in the range of 100–300 nm in diameter. NMV isolated from *N*-formylmethionine-leucyl-phenylalanine (fMLP)-stimulated peripheral blood neutrophils were further characterized using flow cytometry. As MV express molecules characteristic of the parent cell, we analyzed the expression of the neutrophil specific surface antigen CD66b, detecting expression in 57.2 ± 18.87% NMV (Figure 1B). In addition, NMV were stained for annexin V (Figure 1C), demonstrating expression of phosphatidylserine on the surface of NMV, as described for other MV preparations [31,32,33]. Furthermore, there was no detectable contamination by CD14-positive MV, confirming negligible contamination with monocyte MV (Figure 1D).

### 2.2. NMV Are Internalised by Human Brain Endothelial Cells

**Figure 2:**
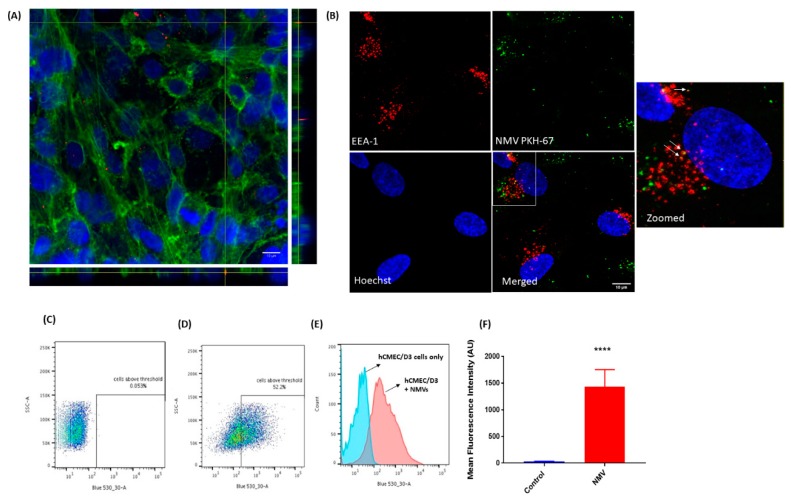
Internalization of NMV by hCMEC/D3. (**A**) NMV interaction was visualized using confocal microscopy. PKH-26- NMV (red) were incubated with hCMEC/D3 for 2 h, fixed and stained (FITC-phalloidin (green) and Topro-3-iodide (blue). Orthogonal views demonstrated the presence of NMV inside the cells. Images were obtained on Zeiss LSM 510 confocal and 63×/1.4 oil immersion lens. (**B**) Confocal analysis of hCMEC/D3 stained for EEA-1 antigen (red), PKH-67 NMV (green) and counter-stained for nuclei (blue). Zeiss LSM 880 with Airyscan with a 63× oil immersion lens was used to obtain the images. Zoomed in image (50%). (**C**) PKH-67- NMV were incubated with hCMEC/D3 for 2 h and cells analyzed for fluorescence by flow cytometry. Trypan blue (1 mg/mL) was added to the sample immediately prior to analysis. Fluorescence intensity of unlabeled endothelial cells was used as a parameter to set the threshold for PKH-67-positivity and to gate-out any cellular autofluorescence. (**D**) hCMEC/D3 incubated with PKH-67 NMV after 2 h (**E**) Representative histogram of florescence intensity of hCMEC/D3 cells with and without NMV. (**F**) Mean Fluorescence Intensity (MFI) was calculated using FlowJo software (FlowJo v10.3). Results represent mean ± SD (*n* = 5, **** *p* < 0.0001, Unpaired *t*-test).

Confocal laser microscopy revealed that fluorescently labelled NMV interacted with a confluent monolayer of hCMEC/D3 cells (Figure 2A). Orthogonal views confirmed the internalization of NMV by brain endothelial cells, detected within 2 h of incubation. To determine whether NMV colocalized with early endosomes, hCMEC/D3 cells were stained with antibodies against early endosome antigen-1 (EEA-1). After 2 h incubation, a proportion of NMV was found to be associated with early endosomes (Figure 2B). The internalization of NMV by hCMEC/D3 was validated and quantified by flow cytometry following the quenching of surface fluorescence using trypan blue. Fluorescently labelled NMV were detected in 48.36 ± 11.37% of brain endothelial cells (Figure 2C–F).

### 2.3. The Internalization of NMV Occurs via Multiple Pathways

**Figure 3:**
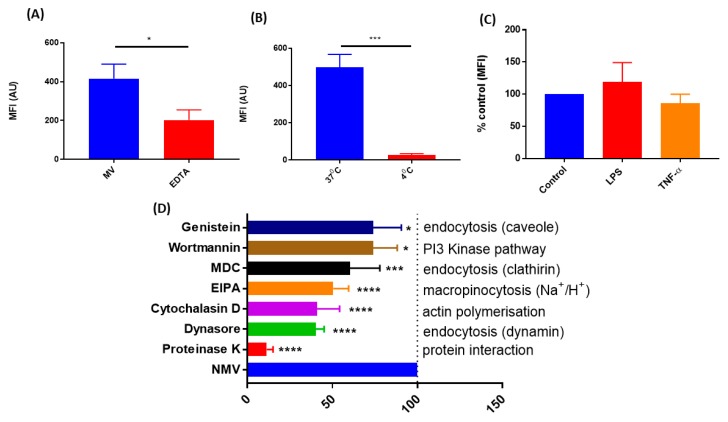
Mechanisms of NMV internalization by hCMEC/D3 cells. (**A**) Co-incubation with EDTA, a metal ion chelator significantly reduced internalization. (**B**) NMV internalization was significantly reduced at 4 °C. (**C**) Pre-incubation with LPS (1 µg/mL) and TNF-α (10 ng/mL) did not affect internalization. (**D**) hCMEC/D3 cells were pre-incubated with pharmacological inhibitors genistein, wortmannin, MDC, EIPA, cytochalasin D, Dynasore, and also proteinase K and PKH-67 labeled NMV were added for 2 h. Data represent 3–5 independent experiments and are presented as the percentage of NMV uptake compared to the positive control with no inhibitors (mean ± SD, One-way ANOVA with Dunnett’s multiple comparison test * *p* < 0.05, *** *p* < 0.001, **** *p* < 0.0001).

Incubation of hCMEC/D3 with NMV at 4 °C significantly decreased the internalization of NMV by 94.65 ± 0.81%, p-0.0001 (Figure 3B), demonstrating that this is an energy-dependent process. NMV internalization was significantly reduced by 51.51 ± 7.219%, p-0.003 (Figure 3A) after the addition of the chelating agent Ethylenediaminetetraacetic Acid (EDTA), indicating that the internalization is metal ion-dependent. Stimulation of hCMEC/D3 with LPS or TNF-α had no effect on NMV internalization (Figure 3C). Pre-treatment with proteinase K, a broad specificity protease, significantly reduced internalization by 88.51 ± 3.619%, p-0.0001 (Figure 3D), suggesting protein–protein interaction is needed for internalization of NMV.

To determine other possible cellular uptake mechanisms responsible for NMV internalization, cells were treated with various pharmacological inhibitors, including: dynasore to inhibit the function of dynamin, cytochalasin D to inhibit actin elongation and the formation of microfilaments, 5-(*N*-Ethyl-*N*-isopropyl)amiloride (EIPA) to block the activity of Na^+^/H^+^ exchangers required for micropinocytosis, monodansylcadaverine (MDC) to inhibit clathrin-mediated endocytosis, and genistein to inhibit caveolin-mediated endocytosis. The largest reduction in internalization was seen with Dynasore (59.79 ± 5.01%, p-0.0001), followed by cytochalasin D (59.05 ± 13.35%, p-0.0001) and EIPA (49.47 ± 9.908%, p-0.0001) (Figure 3D). MDC significantly reduced internalization by 39.55 ± 17.51%, p-0.0004, while genistein and wortmannin had a moderate effect (25.83 ± 14.08%, p-0.0374 and 25.95 ± 16.61%, p-0.0229, respectively). Varying levels of internalization inhibition resulting from targeting these differing pathways suggests that NMV uptake occurs via multiple pathways.

### 2.4. Internalization of NMV Impacts the Transcriptomic Profile of Brain Endothelial Cells

**Figure 4:**
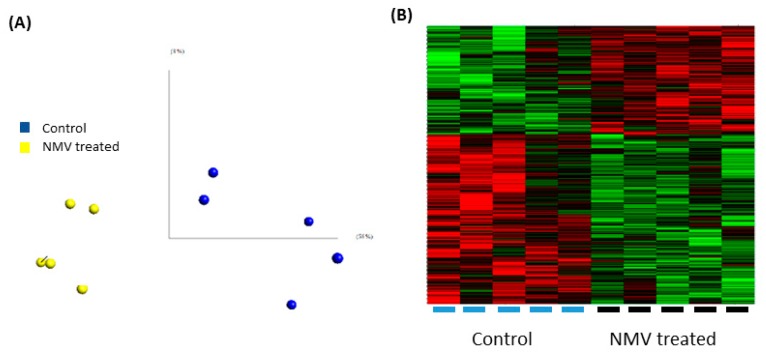
Internalization of NMV significantly impacts the transcriptome of hCMEC/D3. (**A**) Principal component analysis of microarray data. (**B**) Heat map depicting up-regulated (red) and down-regulated (green) gene expression changes (FC ≥ 1.2, *p* ≤ 0.05), 932 genes were significantly differentially expressed (363 up-regulated genes and 569 down-regulated genes). Blue bars represent untreated BMEC samples while the black bars represent BMEC treated with NMV.

While NMV are internalized after 2 h, the time to impact mRNA expression is likely longer. Therefore transcriptomic analysis of hCMEC/D3 after 24 h incubation with NMV was performed using Human Genome U133 Plus 2.0 Arrays, which contain at least one probe for each gene and recognize over 39,000 genes. Principal component analysis confirmed there were no sample outliers (Figure 4A) and QC were within an acceptable range. As shown in the heat map (Figure 4B), the internalization of NMV significantly altered the gene expression profile of hCMEC/D3.

After setting the parameters as fold change ≥ 1.2, *p* value ≤ 0.05, 932 genes were significantly differentially expressed (363 upregulated and 569 downregulated) (GEO public database accession code GSE137111). Functional grouping analysis with the highest stringency setting identified dysregulated genes that were associated with TJ proteins, ubiquitin-mediated proteolysis, vesicular transport and metal cluster binding (Table 1).

Kyoto encyclopedia of genes and genomes or KEGG pathway analysis in Database for Annotation Visualization and Integrated Discovery (DAVID) and IMPaLA identified dysregulated pathways including ubiquitin mediated proteolysis, SNARE mediated vesicular transport and the P38 pathway (Table 2), demonstrating NMV significantly affect the gene expression of major pathways involved in cellular functions in brain endothelial cells.

### 2.5. NMV Increase the Permeability and Decrease the Transendothelial Electrical Resistance of hCMEC/D3

**Figure 5:**
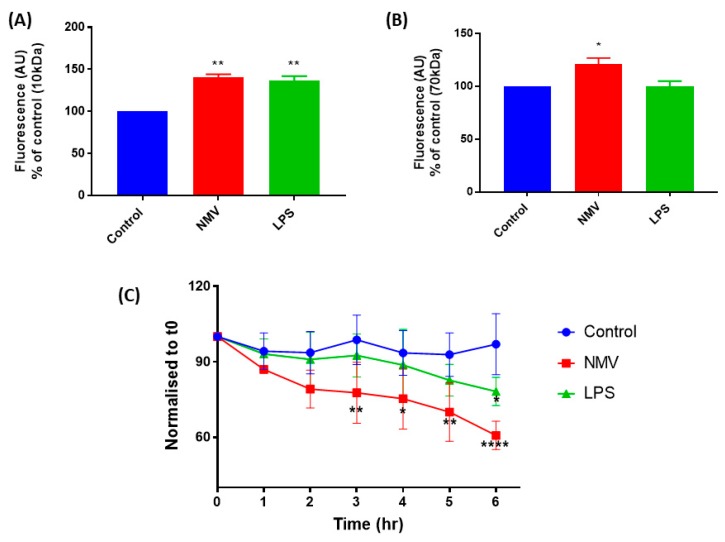
NMV significantly increase the permeability and decrease the electrical resistance of a hCMEC/D3 monolayer. NMV significantly increase the flux of (**A**) 10 kDa dextran and (**B**) 70 kDa dextran across a confluent monolayer of hCMEC/D3. (**C**) NMV significantly decrease TEER within 3 h. Data represent 3 independent experiments, expressed as mean ± SD. Statistical significance was measured using one-way ANOVA, * *p* < 0.05, ** *p* < 0.01, **** *p* < 0.0001.

As the microarray data indicated that the internalization of NMV impacts genes associated with vascular integrity, we sought to validate this finding by assessing the effect of NMV on the permeability of a confluent monolayer of hCMEC/D3. To study the impact of NMV on paracellular leakage, 10 kDa and 70 kDa FITC-dextran were employed to investigate the effect of NMV on the paracellular transport of small and large molecules, respectively. When the paracellular flux of 10 kDa was investigated, there was a significant increase in flux of 10 kDa FITC-dextran to the basolateral chamber (40.8 ± 3.3%, p-0.0024). LPS, which disrupts the endothelial monolayer [34,35], was used as a positive control and significantly increased the flux of 10 kDa FITC-dextran (36.6 ± 5.3%, p-0.0034) (Figure 5A). Similarly, paracellular flux of 70 kDa FITC-dextran was significantly increased when NMV were present (20.9 ± 4.8%, p-0.0272) (Figure 5B), whilst LPS had no significant effect (Figure 5B).

In addition to assessing the effect of NMV on dextran permeability, we also measured TEER, as a quantitative measure of barrier integrity. The internalization of NMV induced a time-dependent effect on the permeability of the cell monolayer in vitro, NMV induced a decrease in the TEER of the confluent monolayer within the first hour and, during the subsequent hours, TEER continued to decrease (Figure 5C). Within 3 h, this decrease in TEER was statistically significant (p-0.0083). LPS was used as a positive control and decreased TEER throughout the time-course (p-0.093). No cell detachment was observed during the 6 h of this experiment. 

## 3. Discussion

The inflammatory signals produced in response to systemic infection may increase BBB permeability and disrupt homeostasis in the CNS leading to neuroinflammation and neuronal dysfunction, exacerbating neurodegenerative processes [5,7,36,37,38]. However, the mechanisms by which peripheral immune activation impacts the CNS remain relatively unknown. In the current study, we demonstrate that NMV are internalized by human brain endothelial cells in vitro, significantly altering the transcriptomic profile and leading to increased monolayer permeability, suggesting a mechanism whereby the peripheral response to infection can induce BMEC dysfunction.

While extravasated neutrophils are not routinely associated with age-related neuropathology and are not usually detected in the brain, individuals with dementia have significantly higher levels of circulating neutrophils than neurological controls [18]. Conflicting reports have suggested that neutrophils from patients with dementia have a reduced immune response [39], while others have indicated neutrophil hyperactivation correlates with the progression of AD [19]. The current study isolated peripheral-blood neutrophils and identified a potential mechanism whereby neutrophils can alter the function of the BMEC. Neutrophils were stimulated with fMLP, a bacterial peptide, to induce MV formation using a well-characterized, standard protocol [25,29,40,41], and NMV were characterized using nanoparticle tracking and annexin V staining, indicating the presence of phosphatidylserine [25,28,30,32]. NMV also expressed CD66b, demonstrating that it originated from neutrophils although heterogeneity in CD66b expression is reported in the literature [25,32,42,43]. CD66b is an important surface marker expressed on neutrophils and is involved in adherence of neutrophils to endothelial cells [44]. The expression of this molecule on the surface of NMV may aid in adhesion to brain endothelial cells, although detailed work is needed to understand how NMV adhere to the cells. 

Uptake of MV originating from other cell types such as monocytes, platelets, and cancer cells has been widely reported [32,43,45,46,47], but to our knowledge, this is the first study to report the internalization of NMV by human brain microvascular endothelial cells. In support of other studies which have demonstrated the interaction between NMV and other endothelial cells, such as HUVEC and HCAEC [29,43], our study using confocal microscopy and flow cytometry confirmed the interaction and subsequent uptake of NMV by hCMEC/D3 cells. It should be noted that while flow cytometry is a standard approach to quantitate the number of cells that have internalized NMV, it does not discriminate between single or multiple NMV uptake. Once internalized, NMV associate with early endosomal compartments, as demonstrated by the co-localization with EEA-1, supporting the role of endocytosis as major route of entry [46,48]. EEA-1 is an early endosomal protein that has been linked with endocytic vesicles before the fusion with early endosomes [49]. However, only a small proportion of NMV co-localized with EEA-1, while the majority were found near to endosomes. Real time tracking of MV through the cell is needed to understand the fate of NMV after they are internalized and localized in the endosome. 

To understand the mechanism of internalization, initially, we investigated whether the process is broadly active or passive. Co-incubation of hCMEC/D3 with NMV at 4 °C almost completely inhibited internalization, demonstrating that uptake is an energy-dependent process [47,50,51]. Furthermore, treatment with proteinase K significantly reduced internalization, demonstrating that uptake likely requires some form of initial surface binding. There are ongoing studies to identify the key proteins present on the surface of MV [52] and further studies to identify which proteins are involved in the recognition of, and binding to, target cells are required.

Uptake of NMV by hCMEC/D3 was partially inhibited by EDTA, a metal ion chelator, suggesting that internalization may be partially calcium-dependent [46,53]. To further understand the contribution of the endocytotic pathway to NMV internalization, we utilized pharmacological inhibitors. Cytochalasin D depolymerizes actin, thereby preventing endocytosis [54], while dynasore is a GTPase inhibitor that reversibly inhibits dynamin activity [55]. Both cytochalasin D and dynamin significantly reduced internalization in hCMEC/D3, supporting the findings of other studies that have shown these compounds reduce internalization of extracellular vesicles in other types of cells [46,48,56]. Notably, both inhibitors did not completely inhibit the internalization of NMV uptake. These results together indicate that NMV uptake is energy-dependent and requires cytoskeletal rearrangement, which are both indicative of endocytic pathways. Furthermore, EIPA also significantly reduced internalization, demonstrating a role for macropinocytosis in uptake, in agreement with the literature [46,50,56]. However, it should be noted that EIPA has an effect on cellular pH, which may also impact the uptake of NMV [57]. Other inhibitors, including MDC, wortmannin (PI3-Akt pathway inhibitor), and genistein, also had moderate effects on internalization. Interestingly, none of the chemical inhibitors completely inhibited internalization, possibly due to the heterogeneous nature of the NMV population. NMV range in size from 100 to 1000 nm in diameter [25,26] and the multiple pathways employed for internalization may suggest the mechanism of uptake of NMV is dependent on their size and surface molecule expression [58,59]. However, further studies are required to fully elucidate these mechanisms.

We further demonstrate that inflammatory stimuli such as LPS and TNF-α did not alter NMV internalization. In response to inflammatory stimuli, BMEC increase their expression of ICAM-1. While blocking ICAM-1 reduces the uptake of extracellular vesicles in dendritic cells and HCAEC [29,60], other studies report blocking ICAM-1/VCAM-1 does not reduce internalization of platelet-derived MV by BMECs [46]. Together, these studies highlight that the origin of MV and their target cells play a significant role in the way that MV are internalized.

Various methods of stimulation have been shown to elicit the formation of NMV that affect other target cells, such as endothelial cells [32,61,62] and macrophages [25,63]. NMV impact the gene expression profile of HUVEC [43], and in the current study, we demonstrate that NMV significantly change the transcriptomic profile of human brain endothelial cells. Specifically, NMV induced dysregulation in expression of tight junction proteins, p38 pathway, and SNARE-mediated vesicular transport. One of the differentially expressed genes, Tumor Protein P53 Inducible Nuclear Protein 1 (*TP53INP1*), is a downstream target for miR155 [64] which is enriched in NMV [29]. Other dysregulated genes, including transforming growth factor β receptor 1 (*TGF-βR1)*, BH3- interacting domain 1 (*BID1*), and mitogen activated protein kinase kinase kinase (*MAP3K*), are involved cellular apoptosis and inflammation and can affect vascular integrity [65,66,67,68].

In support of our transcriptomic data, functional studies confirmed that NMV significantly decrease the integrity and significantly increase the permeability of a confluent monolayer of hCMEC/D3, supporting studies which have demonstrated increased disruption of the BBB by MV isolated from the plasma in vitro [69] and exosomes in vivo [70]. While our results confirm previous findings that hCMEC/D3 have a TEER ranging from 20 to 30 Ω/cm^2^ [71,72], it should be noted that this does not reflect the TEER of the BBB in vivo, which ranges between 1500 and 2000 Ω/cm^2^ [73]. Many methods have been used to increase the TEER in hCMEC/D3, including co-culturing with astrocytes [74] and growing them under sheer stress [75], which increases TEER slightly. Nonetheless, hCMEC/D3 is a robust, commonly utilized model of the BBB [72,76,77,78].

Systemic inflammation is known to modulate BBB function, and MV have been shown to increase endothelial permeability [79,80]. When the BBB is disturbed, the flux of tracer molecules increases through the cell monolayer [81], thereby allowing us to utilize this effect to study the changes in permeability. Here we report that NMV significantly increase the flux of FITC-dextran (both 10 kDa and 70 kDa) through a confluent monolayer of hCMEC/D3. The change in flux of 10 kDa FITC-dextran was greater than that for 70 kDa, suggesting that NMV have a greater effect on the flux of smaller molecules through the monolayer. Notably, most cytokines have small molecular weights [82] and increased flux of cytokines across the BBB can modulate the neuroinflammatory response [83,84].

In summary, we have shown that the internalization of NMV significantly impacts the transcriptomic profile of hCMEC/D3 cells, including the dysregulation of genes associated with tight junctions and vesicular transport, and significantly increases the permeability and decreases the TEER of a monolayer of brain endothelial cells. This internalization process is energy-dependent and uses multiple endocytic pathways. NMV are being increasingly studied as therapeutic targets. Neutrophils exhibit considerable plasticity and are responsive to bidirectional signaling [85], therefore it may be possible to modulate the peripheral immune response, in particular, neutrophils, to influence therapeutic outcomes in AD. Understanding the effects of NMV on the BMEC could identify a potential mechanism linking systemic inflammation and accelerated cognitive decline in dementia patients, however further work is required to assess the impact of NMV on the BMEC in a relevant animal model in vivo and to characterize the content of NMV. In conclusion, this study provides novel insights into the role of NMV in BMEC dysfunction.

## 4. Materials and Methods

### 4.1. Cell Culture

hCMEC/D3 cells were obtained from Cedarlane, Canada and were cultured in EBM-2 medium with 2.5% FBS and supplemented with vascular endothelial growth factor (VEGF), insulin-like growth factor (IGF-1), human epidermal growth factor (hFGF), human basic fibroblast growth factor (hFGF), hydrocortisone, and ascorbic acid (all provided in EGM-2 MV bulletkit, Lonza, Basel, Switzerland). Cells were cultured on Collagen type-I (Sigma, Gillingham, UK) coated plates. All experiments were carried out between passage 27–35.

### 4.2. Neutrophil-Derived Microvesicle Isolation

Venous blood was obtained from consenting healthy volunteers over the age of 18 with no known medical conditions following ethical approval from the University of Sheffield Research Ethics Committee (reference No: SMBRER310, Date of approval, 1/05/2014). Peripheral blood neutrophils were isolated based on the protocol by Timar et al. [86] with minor modifications. Briefly, peripheral venous blood was collected in tubes containing 3.8% sodium citrate and was separated by density gradient centrifugation using Histopaque-1077 (Cambridge Bioscience, Cambridge, UK). Red blood cells were lysed, and isolated neutrophils were stimulated with the bacterial-derived peptide fMLP (10 µmol/L, Sigma) for 1 h (37 °C in 5% CO_2_). Neutrophils and large cell debris were pelleted by centrifugation (500× *g* for 5 min followed by 1500× *g*, 5 min respectively). A sample was taken for cytospin to check for any cellular contamination. The supernatant was centrifuged at 20,000× *g* for 30 min to pellet NMV. The pelleted NMV were resuspended in 0.2-µm sterile-filtered PBS. The number of NMV from each isolation was determined using a BD LSRII flow cytometer (Becton Dickinson, Wokingham, UK). The settings were standardized on forward-scatter and side-scatter gates using Megamix beads of various sizes (0.5, 0.9, 3 µm, BioCytex, Marseille, France). NMV were quantified using Sphero™AccuCount beads (Saxon Europe, Kelso, UK). The flow cytometer was set to count 1000 beads and the concentration of NMV in the sample was calculated using the manufacturer’s instructions.

### 4.3. Flow Cytometric Analysis

NMV were resuspended in PBS and incubated with either FITC-Annexin V (according to manufacturer’s instructions, Biolegend, London, UK), mouse anti-human CD66b (1 µg/mL, Clone: G10F5, BD Bioscience), CD14 (0.75 µg/ ml, Clone: M5E2, BD Bioscience) fluorescently-conjugated antibodies or appropriate isotype controls for 45 min in the dark at 4 °C. Labeling was assessed using a BD LSRII flow cytometer (Becton Dickinson) and data was analyzed using FlowJo software (FlowJo v10.3; Treestar Inc, Ashland, OR, USA).

### 4.4. Nanoparticle Tracking Analysis

Size distribution of NMVs was determined using nanoparticle tracking analysis. The ZetaView (Particle Metrix, Meerbusch, Germany) was calibrated using 110 nm polystyrene beads (Thermofisher, Runcorn, UK). NMVs were diluted in milliQ water and loaded into the cell. The frame rate was set to 3.75 frames/second and shutter speed of 70. Measurements were taken at 11 positions in the cell, with 2 cycles of each position. Data were then analyzed using Particle Metrix software (ZetaView 8.03.08.03) and Microsoft Excel 2010 (Microsoft Corp., Washington, DC, USA).

### 4.5. Neutrophil Microvesicle Internalization by Endothelial Cells

NMV were fluorescently labeled with PKH-26 or PKH-67 (Sigma) according to the manufacturer’s instructions. NMV were pelleted by centrifugation (20,000× *g*, 30 min). Fluorescently-labeled NMV (300 NMV/µL) were added to hCMEC/D3 cells plated on collagen type-I coated 24-well plates (30,000 cells/well and grown to confluence) and incubated at 37 °C for 2 h. For flow cytometric analysis, cells were washed, trypsinised, and resuspended in PBS. 100 µL of trypan blue (1 mg/mL) was added to each sample immediately prior to analysis on the flow cytometer to ensure the fluorescent signal detected was only from internalized NMV [87]. Changes in mean fluorescence intensity and % positive cells were analyzed using FACSDiva acquisition software version 8 (Becton Dickinson) and FlowJo analysis software v10.3 (Treestar Inc, Ashland, OR, USA.

To determine PKH-26 localization, the cells were fixed with 4% paraformaldehyde for 10 min, washed twice, and permeabilized with 0.1% Triton-X for 3 min. The cells were washed three times and non-specific binding was blocked using 3% BSA for 30 min. Actin filaments were stained using FITC-Phalloidin (1:200) and nuclei were counterstained using TOPRO-3-iodide (1 µg/mL, Life Technologies, Paisley, UK).

For the co-localization experiment, hCMEC/D3 cells were stained using antibodies to EEA-1 antigen (SC-365652, 5µg/mL, SantaCruz Biotechnology, Heidelberg, Germany) and nuclei were counterstained using Hoechst nuclear dye (1 µg/mL, Sigma). Localization of NMV was visualized using a Zeiss KSM 880 with an Airyscan confocal microscope and images were obtained using Zeiss Leica Zen imaging software (Leica, Milton Keynes, UK).

### 4.6. Analysis of Internalization Pathways

hCMEC/D3 cells were plated as described above. To determine if inflammatory stimuli such as TNF-α or LPS influence the rate of internalization, cells were preincubated with TNF-α (10 ng/mL) or LPS (1 µg/mL) 16 h before adding NMV.

For inhibition experiments, EIPA (50 µM), EDTA (0.1 µM), or cytochalasin D (1 µM) were added to the wells 30 min prior to NMV addition, while monodansylcadaverine (MDC) (150 µM) or Dynasore (1 µM) were added 1 h prior. Cells were preincubated for 1 h with genistein (100 µM) and wortmannin (1 µM), which removed by aspiration before the addition of NMV. For proteinase K (150 ug/mL), cells were pre-incubated for 30 min and washed once with PBS before adding NMV. The mean fluorescence intensity was measured by flow cytometry as described above.

### 4.7. RNA Extraction and Amplification

hCMEC/D3 cells were incubated with NMV for 24 h and RNA was extracted using the Direct-zol RNA kits (Cambridge Bioscience), according to the manufacturer’s instructions. The quantity and quality of the extracted RNA were determined using Nanodrop 1000 spectrophotometer (Thermo Fisher Scientific, Altrincham, UK) and a 2100 Bioanalyzer (Agilent, Stockport, UK).

### 4.8. Microarray Amplification and Microarray Hybridization

For RNA amplification, the GeneChip 3′ IVT Express amplification protocol (Thermo Fisher Scientific) was used. Briefly, 100 ng of total RNA was annealed to an oligo-d(T) primer with a T7 polymerase binding site. After the generation of double-stranded complementary DNA (cDNA), copy RNA (cRNA) was transcribed, which acted as the RNA template for second round amplification. At the end of this round, biotin-labeled cRNA was prepared using the Affymetrix Gene Chip in vitro transcription labelling kit (Affymetrix). The RNA quality was assessed after the clean-up of biotin-labeled cRNA using Agilent Bioanalyser 2100 (Agilent) 12.5ug of labeled antisense RNA was fragmented and 11 µg applied to HGU133 Plus 2.0 gene microarrays and hybridized over 16 h at 45 °C in a rotating oven at 60 rpm. Post-hybridization washing and sample staining was carried out using FluidicStation400 (Thermo Fisher Scientific) and the Gene Chip Operating System (Thermo Fisher Scientific) and was scanned using a GC3000 7G scanner (Thermo Fisher Scientific).

### 4.9. Microarray Analysis

The Robust Multi-array Average (RMA) was used to determine intensity distribution and eliminate sample outliers [88]. Microarray analysis was carried out using Qlucore Omics Explorer (Qlucore, Lund, Sweden) and setting the parameters of significantly differentially expressed genes as *p* ≤ 0.05 and fold change of minimum 1.2. Functional analysis was performed using the Database for Annotation Visualization and Integrated Discovery (DAVID) and PANTHER to group the genes according to their function and functional pathways.

### 4.10. Permeability Assay

To assess monolayer permeability, hCMEC/D3 cells were cultured on 24 well transwell inserts (0.4 µm pores, PET, Starsstead) to confluence for 4 days. To measure the barrier integrity, FITC-dextran of 10 kDa and 70 kDa (1 mg/mL, Sigma) was added to the apical chamber in phenol red-free media. Samples (100 µL) were taken from the basolateral chamber at times 0, 6, and 24 h. The equivalent volume of phenol red-free medium was added back to the chamber to compensate for the reduction in volume due to sampling. Transwell inserts without cells were included as controls for membrane interference. Fluorescence was measured using a plate reader with excitation at 492 nm and emission at 520 nm.

### 4.11. Transendothelial Electrical Resistance (TEER)

Transwell inserts were coated with collagen-I were used to seed hCMEC/D3 cells grown to confluence. TEER was measured in triplicates using the STX01 electrode and Millicell- ERS Volt-Ohmmeter. NMV (300/µL) were added to the apical side and three readings were taken every hour for 6 h, and the average was used to calculate TEER. LPS (1 µg/mL) was used as a positive control.

### 4.12. Statistical Analysis

All statistical analysis was performed using GraphPad Prism version 7.00 (Graphpad software, San Diego, CA, USA). Results are presents as mean ± standard deviation unless otherwise stated. Analyses used are indicated in each figure legend.

## Figures and Tables

**Table 1:** Functional grouping analysis of microarray data using Database for Annotation Visualization and Integrated Discovery (DAVID).

Functional Group	*p*-Value	Number of Genes
Tight junction proteins	0.0062	6
Vesicle mediated transport	0.0074	19
Protein transport	0.0019	18
RNA localization	0.0011	9
Metal cluster binding	0.032	4
FY-rich terminals	0.0043	3

**Table 2:** KEGG pathway analysis of microarray data using DAVID and IMPaLA.

Pathway	*p*-Value
Ubiquitin-mediated proteolysis	0.034
SNARE-mediated vesicular transport	0.0163
P38 pathway/Regulation of SMAD2/3 signaling	0.0191
Coenzyme B biosynthesis	0.0209
Gap junction degradation	0.0324

## Abbreviations

AD	Alzheimer’s Disease
BBB	Blood brain barrier
BMEC	Brain microvascular endothelial cells
CNS	Central nervous system
EDTA	Ethylenediaminetetraacetic Acid
EEA-1	Early endosomal antigen-1
EIPA	5-(N-Ethyl-N-isopropyl)amiloride
fMLP	N-formylmethionine-leucyl-phenylalanine
HCAEC	Human coronary arterial endothelial cells
HUVEC	Human umbilical vein endothelial cells
ICAM-1	Intercellular adhesion molecule-1
LPS	Lipopolysachharide
MDC	Monodansylcadaverine
MFI	Mean Fluorescent Intensity
MV	Microvesicles
NMV	Neutrophil derived microvesicles
NVU	Neuro vascular unit
TEER	Tranendothelial electrical resistance
TJ	Tight junction
VaD	Vascular Dementia

## References

[1] Cai Z., Qiao P.-F., Wan C.-Q., Cai M., Zhou N.-K., Li Q. (2018). Role of Blood-Brain Barrier in Alzheimer’s Disease. J. Alzheimers Dis..

[2] Wang F., Cao Y., Ma L., Pei H., Rausch W.D., Li H. (2018). Dysfunction of Cerebrovascular Endothelial Cells: Prelude to Vascular Dementia. Front. Aging Neurosci..

[3] Minagar A., Alexander J.S. (2003). Blood-brain barrier disruption in multiple sclerosis. Mult. Scler..

[4] Dantzer R., O’Connor J.C., Freund G.G., Johnson R.W., Kelley K.W. (2008). From inflammation to sickness and depression: When the immune system subjugates the brain. Nat. Rev. Neurosci..

[5] Cunningham C., Hennessy E. (2015). Co-morbidity and systemic inflammation as drivers of cognitive decline: New experimental models adopting a broader paradigm in dementia research. Alzheimers Res. Ther..

[6] Lim A., Krajina K., Marsland A.L. (2013). Peripheral Inflammation and Cognitive Aging. Modern Trends in Psychiatry.

[7] Holmes C., Cunningham C., Zotova E., Woolford J., Dean C., Kerr S., Culliford D., Perry V.H. (2009). Systemic inflammation and disease progression in Alzheimer disease. Neurology.

[8] Gupta A., Iadecola C. (2015). Impaired Aβ clearance: A potential link between atherosclerosis and Alzheimer’s disease. Front. Aging Neurosci..

[9] Yarchoan M., Xie S.X., Kling M.A., Toledo J.B., Wolk D.A., Lee E.B., Van Deerlin V., Lee V.M.-Y., Trojanowski J.Q., Arnold S.E. (2012). Cerebrovascular atherosclerosis correlates with Alzheimer pathology in neurodegenerative dementias. Brain.

[10] Pugazhenthi S., Qin L., Reddy P.H. (2017). Common neurodegenerative pathways in obesity, diabetes, and Alzheimer’s disease. Biochim. Biophys. Acta Mol. Basis Dis..

[11] Ide M., Harris M., Stevens A., Sussams R., Hopkins V., Culliford D., Fuller J., Ibbett P., Raybould R., Thomas R. (2016). Periodontitis and Cognitive Decline in Alzheimer’s Disease. PLoS ONE.

[12] Qin L., Wu X., Block M.L., Liu Y., Breese G.R., Hong J.-S., Knapp D.J., Crews F.T. (2007). Systemic LPS causes chronic neuroinflammation and progressive neurodegeneration. Glia.

[13] Semmler A., Okulla T., Sastre M., Dumitrescu-Ozimek L., Heneka M.T. (2005). Systemic inflammation induces apoptosis with variable vulnerability of different brain regions. J. Chem. Neuroanat..

[14] Nishioku T., Dohgu S., Takata F., Eto T., Ishikawa N., Kodama K.B., Nakagawa S., Yamauchi A., Kataoka Y. (2009). Detachment of brain pericytes from the basal lamina is involved in disruption of the blood-brain barrier caused by lipopolysaccharide-induced sepsis in mice. Cell. Mol. Neurobiol..

[15] Huang C., Irwin M.G., Wong G.T.C., Chang R.C.C. (2018). Evidence of the impact of systemic inflammation on neuroinflammation from a non-bacterial endotoxin animal model. J. Neuroinflammation.

[16] Hoogland I.C.M., Houbolt C., van Westerloo D.J., van Gool W.A., van de Beek D. (2015). Systemic inflammation and microglial activation: Systematic review of animal experiments. J. Neuroinflammation.

[17] Zenaro E., Pietronigro E., Della Bianca V., Piacentino G., Marongiu L., Budui S., Turano E., Rossi B., Angiari S., Dusi S. (2015). Neutrophils promote Alzheimer’s disease-like pathology and cognitive decline via LFA-1 integrin. Nat. Med..

[18] Shad K.F., Aghazadeh Y., Ahmad S., Kress B. (2013). Peripheral markers of Alzheimer’s disease: Surveillance of white blood cells. Synapse.

[19] Dong Y., Lagarde J., Xicota L., Corne H., Chantran Y., Chaigneau T., Crestani B., Bottlaender M., Potier M.-C., Aucouturier P. (2018). Neutrophil hyperactivation correlates with Alzheimer’s disease progression. Ann. Neurol..

[20] Muralidharan-Chari V., Clancy J.W., Sedgwick A., D’Souza-Schorey C. (2010). Microvesicles: Mediators of extracellular communication during cancer progression. J. Cell Sci..

[21] Raposo G., Stoorvogel W. (2013). Extracellular vesicles: Exosomes, microvesicles, and friends. J. Cell Biol..

[22] Nieuwland R., Berckmans R.J., McGregor S., Boing A.N., Romijn F.P., Westendorp R.G., Hack C.E., Sturk A. (2000). Cellular origin and procoagulant properties of microparticles in meningococcal sepsis. Blood.

[23] Prakash P.S., Caldwell C.C., Lentsch A.B., Pritts T.A., Robinson B.R.H. (2012). Human microparticles generated during sepsis in patients with critical illness are neutrophil-derived and modulate the immune response. J. Trauma Acute Care Surg..

[24] Chironi G., Simon A., Hugel B., Del Pino M., Gariepy J., Freyssinet J.-M., Tedgui A. (2006). Circulating leukocyte-derived microparticles predict subclinical atherosclerosis burden in asymptomatic subjects. Arterioscler. Thromb. Vasc. Biol..

[25] Gasser O., Schifferli J.A. (2004). Activated polymorphonuclear neutrophils disseminate anti-inflammatory microparticles by ectocytosis. Blood.

[26] Hess C., Sadallah S., Hefti A., Landmann R., Schifferdi J.A. (1998). Ectosomes released by human neutrophils are specialized functional units. Mol. Immunol..

[27] Hong Y., Eleftheriou D., Hussain A.A.K., Price-Kuehne F.E., Savage C.O., Jayne D., Little M.A., Salama A.D., Klein N.J., Brogan P.A. (2012). Anti-neutrophil cytoplasmic antibodies stimulate release of neutrophil microparticles. J. Am. Soc. Nephrol..

[28] Jaffe E.A., Nachman R.L., Becker C.G., Minick C.R. (1973). Culture of Human Endothelial Cells Derived from Umbilical Veins. Identification by Morphologic and Immunologic Criteria. J. Clin. Invest..

[29] Gomez I., Ward B., Souilhol C., Recarti C., Ariaans M., Johnston J., Burnett A., Mahmoud M., Luong L.A., West L. (2018). Neutrophil microvesicles drive atherosclerosis by delivering miR-155 to atheroprone endothelium. bioRxiv.

[30] Dalli J., Norling L.V., Renshaw D., Cooper D., Leung K.-Y., Perretti M. (2008). Annexin 1 mediates the rapid anti-inflammatory effects of neutrophil-derived microparticles. Blood.

[31] Rhys H.I., Dell’Accio F., Pitzalis C., Moore A., Norling L.V., Perretti M. (2018). Neutrophil Microvesicles from Healthy Control and Rheumatoid Arthritis Patients Prevent the Inflammatory Activation of Macrophages. EBioMedicine.

[32] Pitanga T.N., de Aragão França L., Rocha V.C.J., Meirelles T., Borges V.M., Gonçalves M.S., Pontes-de-Carvalho L.C., Noronha-Dutra A.A., dos-Santos W.L.C. (2014). Neutrophil-derived microparticles induce myeloperoxidase-mediated damage of vascular endothelial cells. BMC Cell Biol..

[33] Gyorgy B., Szabo T.G., Pasztoi M., Pal Z., Misjak P., Aradi B., Laszlo V., Pallinger E., Pap E., Kittel A. (2011). Membrane vesicles, current state-of-the-art: Emerging role of extracellular vesicles. Cell. Mol. Life Sci..

[34] Wong D., Dorovini-Zis K., Vincent S.R. (2004). Cytokines, nitric oxide, and cGMP modulate the permeability of an in vitro model of the human blood–brain barrier. Exp. Neurol..

[35] Gaillard P.J., de Boer A., Bert G., Breimer D.D. (2003). Pharmacological investigations on lipopolysaccharide-induced permeability changes in the blood–brain barrier in vitro. Microvasc. Res..

[36] Marsland A.L., Gianaros P.J., Kuan D.C.-H., Sheu L.K., Krajina K., Manuck S.B. (2015). Brain morphology links systemic inflammation to cognitive function in midlife adults. Brain. Behav. Immun..

[37] King E., O’Brien J.T., Donaghy P., Morris C., Barnett N., Olsen K., Martin-Ruiz C., Taylor J.-P., Thomas A.J. (2018). Peripheral inflammation in prodromal Alzheimer’s and Lewy body dementias. J. Neurol. Neurosurg. Psychiatry.

[38] Teixeira F.B., Saito M.T., Matheus F.C., Prediger R.D., Yamada E.S., Maia C.S.F., Lima R.R. (2017). Periodontitis and Alzheimer’s Disease: A Possible Comorbidity between Oral Chronic Inflammatory Condition and Neuroinflammation. Front. Aging Neurosci..

[39] Le Page A., Dupuis G., Frost E.H., Pawelec G.P., Witkowski J.M., Larbi A., Fülöp T. (2017). Role of the Innate Immune Response in the Progression of Alzheimer’s Disease. Innov. Aging.

[40] Pliyev B.K., Kalintseva M.V., Abdulaeva S.V., Yarygin K.N., Savchenko V.G. (2014). Neutrophil microparticles modulate cytokine production by natural killer cells. Cytokine.

[41] Nolan S., Dixon R., Norman K., Hellewell P., Ridger V. (2008). Nitric Oxide Regulates Neutrophil Migration through Microparticle Formation. Am. J. Pathol..

[42] Porro C., Lepore S., Trotta T., Castellani S., Ratclif L., Battaglino A., Di Gioia S., Martínez M.C., Conese M., Maffione A.B. (2010). Isolation and characterization of microparticles in sputum from cystic fibrosis patients. Respir. Res..

[43] Dalli J., Montero-Melendez T., Norling L.V., Yin X., Hinds C., Haskard D., Mayr M., Perretti M. (2013). Heterogeneity in neutrophil microparticles reveals distinct proteome and functional properties. Mol. Cell. Proteomics.

[44] Kim D., Haynes C.L. (2013). On-chip evaluation of neutrophil activation and neutrophil-endothelial cell interaction during neutrophil chemotaxis. Anal. Chem..

[45] Li J., Zhang Y., Liu Y., Dai X., Li W., Cai X., Yin Y., Wang Q., Xue Y., Wang C. (2013). Microvesicle-mediated transfer of microRNA-150 from monocytes to endothelial cells promotes angiogenesis. J. Biol. Chem..

[46] Faille D., El-Assaad F., Mitchell A.J., Alessi M.-C., Chimini G., Fusai T., Grau G.E., Combes V. (2012). Endocytosis and intracellular processing of platelet microparticles by brain endothelial cells. J. Cell. Mol. Med..

[47] Kawamoto T., Ohga N., Akiyama K., Hirata N., Kitahara S., Maishi N., Osawa T., Yamamoto K., Kondoh M., Shindoh M. (2012). Tumor-Derived Microvesicles Induce Proangiogenic Phenotype in Endothelial Cells via Endocytosis. PLoS ONE.

[48] Fitzner D., Schnaars M., van Rossum D., Krishnamoorthy G., Dibaj P., Bakhti M., Regen T., Hanisch U.-K., Simons M. (2011). Selective transfer of exosomes from oligodendrocytes to microglia by macropinocytosis. J. Cell Sci..

[49] Wilson J.M., de Hoop M., Zorzi N., Toh B.H., Dotti C.G., Parton R.G. (2000). EEA1, a tethering protein of the early sorting endosome, shows a polarized distribution in hippocampal neurons, epithelial cells, and fibroblasts. Mol. Biol. Cell.

[50] Schneider D.J., Speth J.M., Penke L.R., Wettlaufer S.H., Swanson J.A., Peters-Golden M. (2017). Mechanisms and modulation of microvesicle uptake in a model of alveolar cell communication. J. Biol. Chem..

[51] Faille D., Combes V., Mitchell A.J., Fontaine A., Juhan-Vague I., Alessi M.-C., Chimini G., Fusai T., Grau G.E. (2009). Platelet microparticles: A new player in malaria parasite cytoadherence to human brain endothelium. FASEB J. Off. Publ. Fed. Am. Soc. Exp. Biol..

[52] Cvjetkovic A., Jang S.C., Konečná B., Höög J.L., Sihlbom C., Lässer C., Lötvall J. (2016). Detailed Analysis of Protein Topology of Extracellular Vesicles–Evidence of Unconventional Membrane Protein Orientation. Sci. Rep..

[53] Yuan D., Zhao Y., Banks W.A., Bullock K.M., Haney M., Batrakova E., Kabanov A. (2017). V Macrophage exosomes as natural nanocarriers for protein delivery to inflamed brain. Biomaterials.

[54] Lamaze C., Fujimoto L.M., Yin H.L., Schmid S.L. (1997). The actin cytoskeleton is required for receptor-mediated endocytosis in mammalian cells. J. Biol. Chem..

[55] Macia E., Ehrlich M., Massol R., Boucrot E., Brunner C., Kirchhausen T. (2006). Dynasore, a cell-permeable inhibitor of dynamin. Dev. Cell.

[56] Kuhn D.A., Vanhecke D., Michen B., Blank F., Gehr P., Petri-Fink A., Rothen-Rutishauser B. (2014). Different endocytotic uptake mechanisms for nanoparticles in epithelial cells and macrophages. Beilstein J. Nanotechnol..

[57] Koivusalo M., Welch C., Hayashi H., Scott C.C., Kim M., Alexander T., Touret N., Hahn K.M., Grinstein S. (2010). Amiloride inhibits macropinocytosis by lowering submembranous pH and preventing Rac1 and Cdc42 signaling. J. Cell Biol..

[58] Sun X.-Y., Gan Q.-Z., Ouyang J.-M. (2017). Size-dependent cellular uptake mechanism and cytotoxicity toward calcium oxalate on Vero cells. Sci. Rep..

[59] Rejman J., Oberle V., Zuhorn I.S., Hoekstra D. (2004). Size-dependent internalization of particles via the pathways of clathrin- and caveolae-mediated endocytosis. Biochem. J..

[60] Morelli A.E., Larregina A.T., Shufesky W.J., Sullivan M.L.G., Stolz D.B., Papworth G.D., Zahorchak A.F., Logar A.J., Wang Z., Watkins S.C. (2004). Endocytosis, intracellular sorting, and processing of exosomes by dendritic cells. Blood.

[61] Mesri M., Altieri D.C. (1998). Endothelial cell activation by leukocyte microparticles. J. Immunol..

[62] Mesri M., Altieri D.C. (1999). Leukocyte microparticles stimulate endothelial cell cytokine release and tissue factor induction in a JNK1 signaling pathway. J. Biol. Chem..

[63] Eken C., Martin P.J., Sadallah S., Treves S., Schaller M., Schifferli J.A. (2010). Ectosomes released by polymorphonuclear neutrophils induce a MerTK-dependent anti-inflammatory pathway in macrophages. J. Biol. Chem..

[64] Zhang H., Goudeva L., Immenschuh S., Schambach A., Skokowa J., Eiz-Vesper B., Blasczyk R., Figueiredo C. (2015). miR-155 is associated with the leukemogenic potential of the class IV granulocyte colony-stimulating factor receptor in CD34^+^ progenitor cells. Mol. Med..

[65] Walshe T.E., Saint-Geniez M., Maharaj A.S.R., Sekiyama E., Maldonado A.E., D’Amore P.A. (2009). TGF-beta is required for vascular barrier function, endothelial survival and homeostasis of the adult microvasculature. PLoS ONE.

[66] Yeretssian G., Correa R.G., Doiron K., Fitzgerald P., Dillon C.P., Green D.R., Reed J.C., Saleh M. (2011). Non-apoptotic role of BID in inflammation and innate immunity. Nature.

[67] Korsmeyer S.J., Wei M.C., Saito M., Weiler S., Oh K.J., Schlesinger P.H. (2000). Pro-apoptotic cascade activates BID, which oligomerizes BAK or BAX into pores that result in the release of cytochrome c. Cell Death Differ..

[68] Winter-Vann A.M., Johnson G.L. (2007). Integrated activation of MAP3Ks balances cell fate in response to stress. J. Cell. Biochem..

[69] Edrissi H., Schock S.C., Hakim A.M., Thompson C.S. (2016). Microparticles generated during chronic cerebral ischemia increase the permeability of microvascular endothelial barriers in vitro. Brain Res..

[70] Yang T., Martin P., Fogarty B., Brown A., Schurman K., Phipps R., Yin V.P., Lockman P., Bai S. (2015). Exosome Delivered Anticancer Drugs Across the Blood-Brain Barrier for Brain Cancer Therapy in Danio Rerio. Pharm. Res..

[71] Eigenmann D.E., Xue G., Kim K.S., Moses A.V., Hamburger M., Oufir M. (2013). Comparative study of four immortalized human brain capillary endothelial cell lines, hCMEC/D3, hBMEC, TY10, and BB19, and optimization of culture conditions, for an in vitro blood-brain barrier model for drug permeability studies. Fluids Barriers CNS.

[72] Weksler B., Romero I.A., Couraud P.-O. (2013). The hCMEC/D3 cell line as a model of the human blood brain barrier. Fluids Barriers CNS.

[73] Butt A.M., Jones H.C., Abbott N.J. (1990). Electrical resistance across the blood-brain barrier in anaesthetized rats: A developmental study. J. Physiol..

[74] Hatherell K., Couraud P.-O., Romero I.A., Weksler B., Pilkington G.J. (2011). Development of a three-dimensional, all-human in vitro model of the blood–brain barrier using mono-, co-, and tri-cultivation Transwell models. J. Neurosci. Methods.

[75] Cucullo L., Couraud P.-O., Weksler B., Romero I.-A., Hossain M., Rapp E., Janigro D. (2008). Immortalized human brain endothelial cells and flow-based vascular modeling: A marriage of convenience for rational neurovascular studies. J. Cereb. Blood Flow Metab..

[76] Papadia K., Markoutsa E., Antimisiaris S.G. (2016). How do the physicochemical properties of nanoliposomes affect their interactions with the hCMEC/D3 cellular model of the BBB?. Int. J. Pharm..

[77] Ni Y., Teng T., Li R., Simonyi A., Sun G.Y., Lee J.C. (2017). TNFα alters occludin and cerebral endothelial permeability: Role of p38MAPK. PLoS ONE.

[78] Markoutsa E., Pampalakis G., Niarakis A., Romero I.A., Weksler B., Couraud P.-O., Antimisiaris S.G. (2011). Uptake and permeability studies of BBB-targeting immunoliposomes using the hCMEC/D3 cell line. Eur. J. Pharm. Biopharm..

[79] Densmore J.C., Signorino P.R., Ou J., Hatoum O.A., Rowe J.J., Shi Y., Kaul S., Jones D.W., Sabina R.E., Pritchard K.A. (2006). Endothelium-derived microparticles induce endothelial dysfunction and acute lung injury. Shock.

[80] Andrews A.M., Lutton E.M., Merkel S.F., Razmpour R., Ramirez S.H. (2016). Mechanical Injury Induces Brain Endothelial-Derived Microvesicle Release: Implications for Cerebral Vascular Injury during Traumatic Brain Injury. Front. Cell. Neurosci..

[81] Tai L.M., Holloway K.A., Male D.K., Loughlin A.J., Romero I.A. (2010). Amyloid-β-induced occludin down-regulation and increased permeability in human brain endothelial cells is mediated by MAPK activation. J. Cell. Mol. Med..

[82] Akdis M., Aab A., Altunbulakli C., Azkur K., Costa R.A., Crameri R., Duan S., Eiwegger T., Eljaszewicz A., Ferstl R. (2016). Interleukins (from IL-1 to IL-38), interferons, transforming growth factor beta, and TNF-alpha: Receptors, functions, and roles in diseases. J. Allergy Clin. Immunol..

[83] Biesmans S., Meert T.F., Bouwknecht J.A., Acton P.D., Davoodi N., De Haes P., Kuijlaars J., Langlois X., Matthews L.J.R., Ver Donck L. (2013). Systemic immune activation leads to neuroinflammation and sickness behavior in mice. Mediators Inflamm..

[84] Henry C.J., Huang Y., Wynne A.M., Godbout J.P. (2009). Peripheral Lipopolysaccharide (LPS) challenge promotes microglial hyperactivity in aged mice that is associated with exaggerated induction of both pro-inflammatory IL-1β and anti-inflammatory IL-10 cytokines. Brain. Behav. Immun..

[85] Mantovani A., Cassatella M.A., Costantini C., Jaillon S. (2011). Neutrophils in the activation and regulation of innate and adaptive immunity. Nat. Rev. Immunol..

[86] Timár C.I., Lőrincz Á.M., Csépányi-Kömi R., Vályi-Nagy A., Nagy G., Buzás E.I., Iványi Z., Kittel Á., Powell D.W., McLeish K.R. (2013). Antibacterial effect of microvesicles released from human neutrophilic granulocytes. Blood.

[87] Sahlin S., Hed J., Runfquist I. (1983). Differentiation between attached and ingested immune complexes by a fluorescence quenching cytofluorometric assay. J. Immunol. Methods.

[88] Zhang L., Miles M.F., Aldape K.D. (2003). A model of molecular interactions on short oligonucleotide microarrays. Nat. Biotechnol..

